# A Comparative Analysis of Microglial and Astrocyte Transplantation in an Amyloid-Induced Rat for Alzheimer’s Disease

**DOI:** 10.3390/ijms27219667

**Published:** 2026-10-28

**Authors:** Loredana Mariana Curecheriu, Bogdan Florin Iliescu, Gabriela Dumitrița Stanciu, Ivona Mazarianu, Andrei Szilagyi, Maria-Raluca Gogu, Mitica Ciorpac, Ioana-Miruna Balmus, Paul Tutu, Loredana Horodincu, Florentina Daraban Bocaneti, Ozana Maria Hritcu, Aurelian-Sorin Pasca, Bogdan Ionel Tamba, Mihaela Dana Turliuc

**Affiliations:** 1 Department of Neurosurgery, Grigore T. Popa University of Medicine and Pharmacy Iasi, 700115 Iasi, Romania; 2 Department of Neurosurgery “Prof. Dr. N. Oblu” Emergency Clinical Hospital, 700361 Iasi, Romania; 3 Advanced Research and Development Center for Experimental Medicine “Prof. Ostin C. Mungiu”—CEMEX, Grigore T. Popa University of Medicine and Pharmacy Iasi, 700115 Iasi, Romania; 4 Faculty of Veterinary Medicine, “Ion Ionescu de la Brad” Iasi University of Life Sciences, Mihail Sadoveanu Alley 8, 700490 Iasi, Romania

**Keywords:** Alzheimer’s disease, microglia, astrocyte, amyloid-beta 1–42, stereotactic transplantation, treatment, cognitive improvement

## Abstract

Alzheimer’s disease (AD) is recognized as a neuroglial disorder in which dysregulated glial responses contribute to cognitive decline. We investigated whether intrahippocampal transplantation of primary glial cells modifies cognitive trajectories in an amyloid-induced rat model of AD. Sprague–Dawley rats received bilateral hippocampal injections of pre-aggregated Aβ_1–42_, followed by transplantation of primary astrocytes (AA) or microglia (AM). Memory was assessed using the Y-maze and novel object recognition (NOR) tests. Body weight and serum IL-6 and TNF-α were monitored for adverse effects, and GFAP, S100, Iba1 and TREM2 immunolabeling was quantified with QuPath. Mixed-effects models showed significant session and group x session effects on Y–maze spatial novelty recognition. Both treated groups improved compared with AD animals, with a stronger late effect in the AM group and a significant group × session × sex interaction. In the NOR test, both treated groups outperformed AD animals at the final session, without differences between them. GFAP (glial fibrillary acidic protein) immunoreactivity was reduced in the AM group but remained elevated in the AA group, while TREM2 increased after microglial and decreased after astrocyte transplantation. No adverse effects were detected in the monitored parameters. Intrahippocampal glial transplantation, particularly with microglia, may influence cognitive trajectories in established amyloid pathology.

## 1. Introduction

Alzheimer’s disease (AD) is the most common neurodegenerative disease, which results from the accumulation of amyloid-beta (Aβ) plaques and the formation of neurofibrillary tangles (NFTs) [1,2,3]. These deposits burden the normal state of the glial cells and cause marked neuroinflammation, synaptic and neuronal loss, making the pathology complex and difficult to treat [4]. While a neuroinflammatory response initially has protective functions by facilitating the clearance of neurotoxic plaques and tangles, prolonged and dysregulated activation can contribute to neurodegeneration [4]. Therefore, approaches focusing exclusively on amyloid have shown limited and variable clinical efficacy, supporting the need to consider broader disease mechanisms beyond plaque pathology.

Glial cells have recently been studied for their critical role in AD pathogenesis and their potential implications for targeted therapy [5,6]. These cells have vital roles in synapse modulation and inflammation clearance, being viewed in neurodegenerative disease as a double-edged sword [7,8]. In the context of neurodegenerative disease, glial cells undergo phenotypic shifts that may initially support clearance, but after chronic activation, they contribute to sustained neuroinflammation, synaptic destabilization and neurodegeneration [9]. Recent studies show that astrocytes and microglia have a clear role in shaping disease progression and impact on cognitive decline [10,11,12].

These observations have led to growing interest in glia-targeted therapeutic approaches.

Previous experimental approaches have explored intracerebroventricular or intrahippocampal delivery of either stem cell-derived progenitors or glial cells, resulting in variable functional outcomes [13,14,15,16]. Treatments have proved to be feasible, with effects on cognition and amyloid load, but the experiments do not allow precise evaluation of region-specific circuit modulation, particularly within the hippocampus, the key structure for memory encoding that is affected early on in AD [17]. Moreover, most studies rely on cross-sectional endpoints, limiting insight into whether interventions modify disease progression or produce transient symptomatic effects [18,19].

The hippocampus represents a key structure in memory-impaired AD. Studies have shown that hippocampal microglia were affected and associated with a faster decline in episodic memory and perceptual speed [20,21]. Therefore, targeted therapy of the glial population within the hippocampus may provide insight into whether cellular intervention can influence functional progression in established pathology [22].

The current study addresses these gaps by investigating the effects of direct stereotactic intrahippocampal transplantation of primary glial cells in an amyloid-induced rat model of AD. This targeted delivery strategy enables controlled integration within the memory-relevant circuit and permits comparative evaluation of transplanted glial cells. The study has a longitudinal design, as the intervention was performed after confirmation of cognitive impairment. To our knowledge, primary microglia and astrocytes have not previously been compared after intrahippocampal transplantation with the same follow-up. This design allows the contribution of each glial cell type to be assessed under identical conditions. We hypothesized that both cell types would improve hippocampus-dependent memory compared with untreated AD animals, and that the effect of microglia would be stronger, given their central role in amyloid recognition and clearance.

## 2. Results

### 2.1. Behavioral Impact

**Figure 1:**
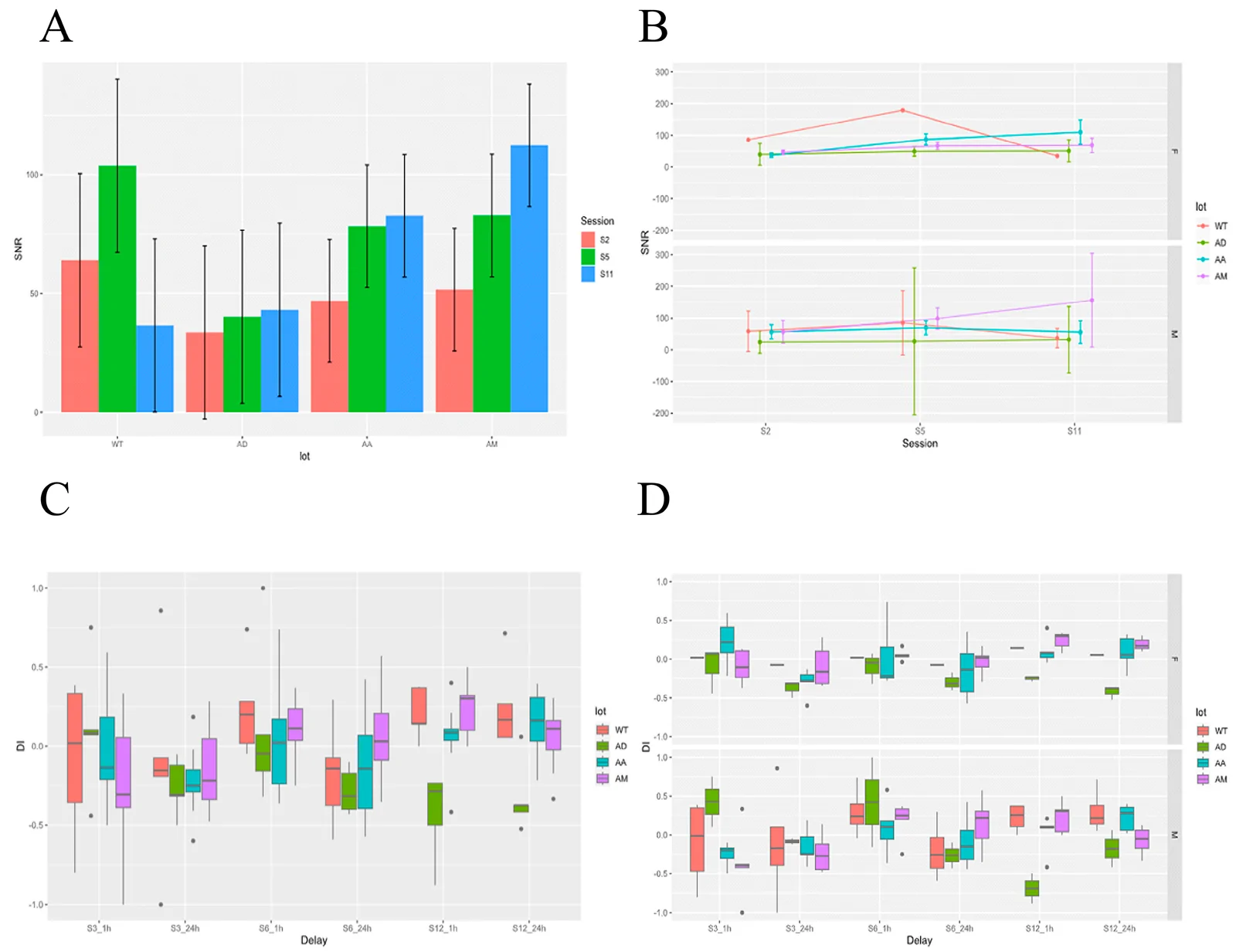
Graphical representation of behavioral analysis. (**A**,**B**) Spatial novelty recognition and exploratory behavior in the Y-maze; A. Spatial novelty recognition (SNR) across groups (WT, AD, AA, AM) at S2, S5, and S11. Bars represent estimated marginal means from the linear mixed-effects model; error bars indicate 95% confidence intervals. B. Sex-stratified longitudinal SNR trajectories across sessions (F, female; M, male). Points represent estimated marginal means; error bars denote 95% confidence intervals. (**C**,**D**) Recognition memory performance in the Novel Object Recognition (NOR) test. C. Discrimination index (DI) across experimental groups (WT, AD, AA, AM) at testing sessions (S3, S6, S12) and retention delays (1 h and 24 h). D. Sex-stratified DI across groups, sessions, and retention delays (F, female; M, male. In (**C**,**D**), box plots show the median and interquartile range, whiskers extended to 1.5× the interquartile range, and dots represent individual outlying values. Confidence intervals rather than standard errors are shown in (**A**,**B**) because they are the direct output of the mixed-effects model and indicate the precision of each estimate.

Linear mixed-effects analysis revealed a significant effect of session (F(2,52) = 4.61, *p* = 0.014) and a significant group × session interaction (F(6,52) = 3.07, *p* = 0.012) for spatial novelty recognition, indicating distinct temporal trajectories across experimental groups. No significant differences were observed between groups at baseline. AD animals failed to show improvement across sessions, whereas both treated groups exhibited significant increases in SNR over time. Notably, the AM group demonstrated the most robust longitudinal improvement, with significantly higher SNR values compared to both WT and AD animals at the final exploration point (S11) (Figure 1A).

Sex-stratified analysis revealed distinct temporal patterns of spatial novelty recognition across experimental groups. In females, both AA and AM treatments induced moderate improvements over time, with comparable magnitudes. In contrast, male animals exhibited a pronounced late increase in SNR in the AM group, exceeding both WT and AD controls at S11. These findings are consistent with the significant group × session × sex interaction identified by linear mixed-effects modeling (Figure 1B).

Total exploration time (TE) during the Y-maze task was analyzed to control potential locomotor or motivational confounds. Linear mixed-effects modeling revealed a significant main effect of experimental group on TE (F(3,26) = 4.94, *p* = 0.0076), while no significant effect of session was observed (F(2,52) = 2.20, *p* = 0.121). The group × session interaction did not reach statistical significance (F(6,52) = 2.19, *p* = 0.058), indicating that differences in exploratory activity across groups were relatively stable over time. Importantly, TE remained within a comparable range across all experimental conditions and sessions, with no evidence of a systematic reduction in exploration that could account for the observed group- and session-dependent differences in spatial novelty recognition performance.

Simple effects analysis for NOR evaluations revealed no significant differences between groups at early time points. In contrast, at the late testing session (S12), AD animals exhibited significantly lower discrimination index values compared to WT controls at both 1 h and 24 h delays (*p* = 0.001 and *p* = 0.003, respectively). Importantly, both treatment groups demonstrated significantly improved recognition memory relative to AD animals at S12, with AA and AM showing higher DI values at 1 h (*p* = 0.004 and *p* < 0.001, respectively) and at 24 h delays (*p* = 0.004 and *p* = 0.022, respectively). No significant differences were observed between the two treatment groups (Figure 1C).

Sex-stratified visualization indicated similar treatment- and delay-dependent effects on recognition index in female and male animals, consistent with the absence of a significant main effect of sex in the mixed-effects analysis (Figure 1D).

Estimated marginal means analysis revealed no significant difference in overall discrimination index between female and male animals, indicating comparable object recognition performance across sexes when averaged across experimental conditions.

Linear mixed-effects analysis of the discrimination index revealed significant main effects of experimental group (F(3,26) = 3.13, *p* = 0.043) and memory delay (F(5,130) = 4.11, *p* = 0.0017), as well as a significant group × delay interaction (F(15,130) = 2.03, *p* = 0.018), indicating that treatment-related differences in object recognition performance were dependent on the retention interval. Post hoc analyses demonstrated that group differences emerged predominantly at longer delays, with AD animals exhibiting reduced discrimination index values, whereas both treatment groups showed significantly improved performance relative to AD at late time points.

### 2.2. Evaluation of Adverse Effects of Transplanted Cells

Body weight changed significantly over time, reflecting normal growth and experimental progression. No significant differences in overall body weight were observed between experimental groups. Although we detected a significant time × group interaction, post hoc analyses revealed no meaningful differences between groups at any time point, indicating that neither disease induction nor glial transplantation affected the monitored parameters.

Log10-transformed IL-6 levels were analyzed using a linear mixed-effects model. A significant main effect of session was observed (F(2,71.9964) = 4.26, *p* = 0.0179), whereas no significant main effect of group (F(3,71.9858) = 1.73, *p* = 0.1675) or group × session interaction (F(6,71.9964) = 1.73, *p* = 0.1253) was detected. A significant group × sex × session interaction was identified (F(20,63) = 3.01, *p* = 0.0005).

For log10-transformed TNF-α, no significant main effect of group (F(3,24) = 1.53, *p* = 0.2319), session (F(2,48) = 2.69, *p* = 0.0779), or group × session interaction (F(6,48) = 0.94, *p* = 0.4735) was observed. The group × sex × session interaction did not reach statistical significance (F(20,32.58) = 1.30, *p* = 0.2469).

### 2.3. Histology and Immunohistochemistry

**Figure 2:**
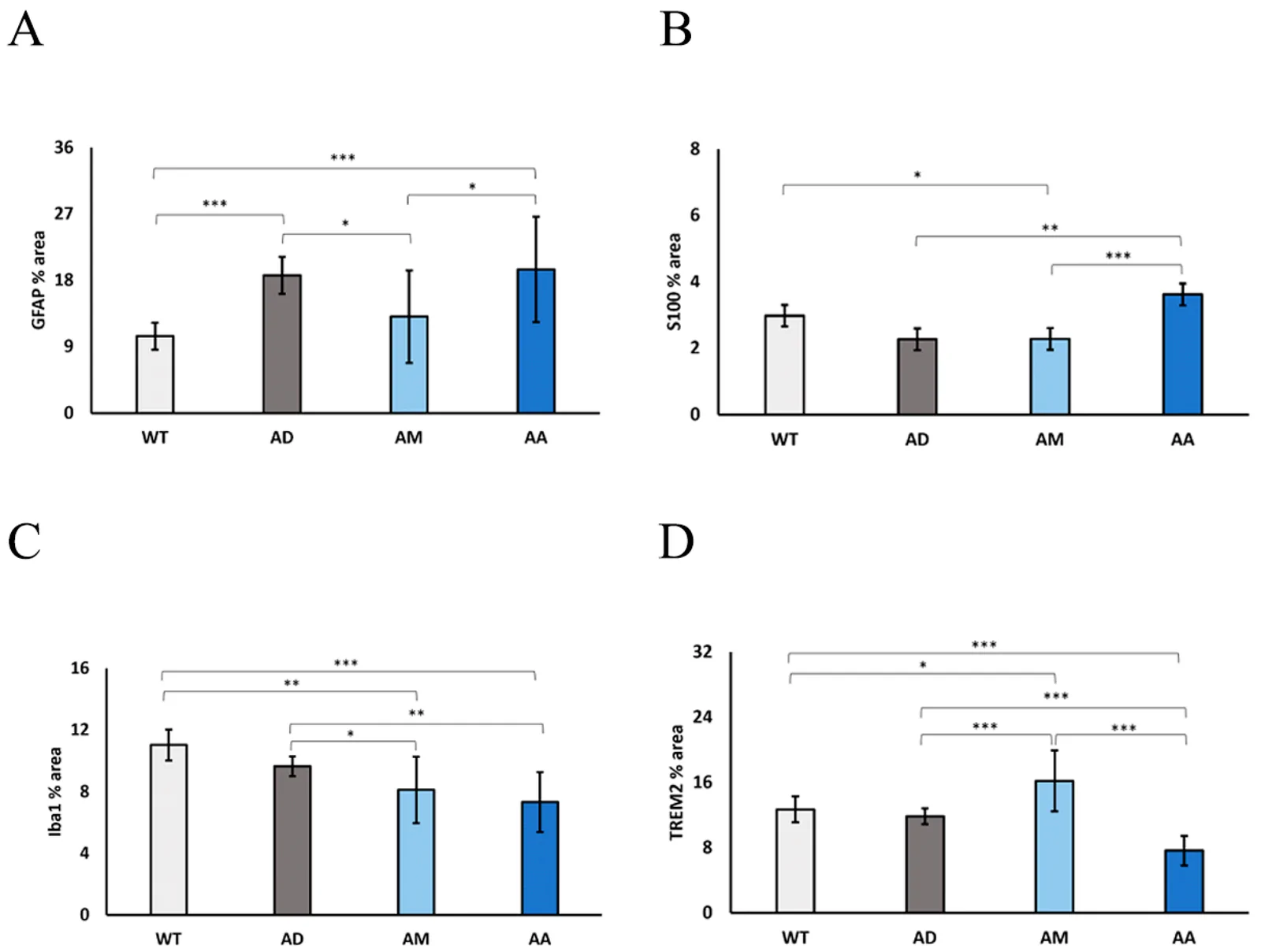
Quantitative immunohistochemical analysis of hippocampal glial markers. Percentage of DAB-positive area for (**A**) GFAP, (**B**) S100, (**C**) Iba1 and (**D**) TREM2 in the WT (wild-type), AD (Aβ_1–42_-injected), AM (AD + microglial transplantation) and AA (AD + astrocyte transplantation) groups. Bars represent mean ± standard deviation; one value per animal, obtained as the mean of the analysed hippocampal sections. Group differences were assessed with Welch’s ANOVA followed by the Games–Howell post hoc test; * *p* < 0.05, ** *p* < 0.01, *** *p* < 0.001.

Welch’s ANOVA for GFAP immunolabeling revealed a highly significant overall difference between groups (W = 17.41, *p* < 0.001). The WT group showed the lowest immunolabeling percentage (10.42 ± 1.84%). Intrahippocampal amyloid injection induced a significantly marked increase in GFAP (18.65 ± 2.50%, *p* < 0.001). The magnitude of the difference between groups indicated a very large effect (g = 5.16), directly reflecting the degree of amyloid-induced reactive astrogliosis. Microglia transplantation significantly reduced reactive astrogliosis in the AM group (13.07 ± 6.26%) compared to the AD group (*p* = 0.02, g = 2.31). In contrast, astrocyte injection maintained a high level of GFAP (19.46 ± 7.15%), with no significant differences compared to the AD group (*p* = 0.97, g = −0.4). A direct comparison between the two therapeutic options demonstrated a significantly greater reduction in GFAP in the AM group compared to the AA group (*p* = 0.02, g = 2.63) (Figure 2A).

The Welch ANOVA test revealed a significant overall variation in S100 levels among the experimental groups (W = 8.98, *p* = 0.001). The positive area for S100 was 2.98 ± 0.42% in the WT group, 2.27 ± 0.56% in the AD group and 2.28 ± 0.78% in the AM group. The reduction compared with WT animals reached statistical significance only in the AM group, whereas the AD and AM groups did not differ from each other (*p* > 0.05). Microglial transplantation therefore did not change S100 immunoreactivity compared with untreated AD animals. In contrast, astrocyte injection led to an increase in the S100-positive area in the AA group (3.62 ± 0.99%), which was significantly higher compared to the AD group (*p* = 0.003, g = 1.38) and the AM group (*p* < 0.001, g = 1.40) (Figure 2B).

The overall differences in total microglial population were significant (W = 14.48, *p* < 0.001). The WT group had the highest Iba1-positive area value (11.04 ± 1.00%). Amyloid injection induced a slight reduction in immunolabeling (9.65 ± 0.64%), but this did not reach statistical significance (*p* = 0.077). Microglia cell therapy (8.12 ± 2.15%) and astrocyte cell therapy (7.32 ± 1.95%) significantly reduced the Iba1-positive area compared to the AD group, with a large effect size (*p* = 0.046, g = 1.09 for microglia; *p* = 0.001, g = 1.74 for astrocytes). No significant differences were observed directly between the two transplanted cell groups (*p* = 0.615) (Figure 2C).

The marker for reactive/phagocytic microglia showed the greatest variation (W = 36.47, *p* < 0.001). The baseline level of TREM2-positive area in the WT group (12.69 ± 1.61%) did not change significantly after amyloid injection (11.84 ± 0.96%, *p* = 0.69). Microglial transplantation significantly increased TREM2 immunoreactivity in the AM group (16.19 ± 3.74%) compared to the AD group (*p* < 0.001, g = 2.37). In contrast, astrocyte transplantation was followed by a marked reduction in TREM2 immunolabeling in the AA group (7.63 ± 1.81 9%) compared to the AD group (*p* < 0.001, g = 3.18). TREM2 immunoreactivity was also significantly higher in the AM group than in the AA group (*p* < 0.001, g = 5.03) (Figure 2D).

**Figure 3:**
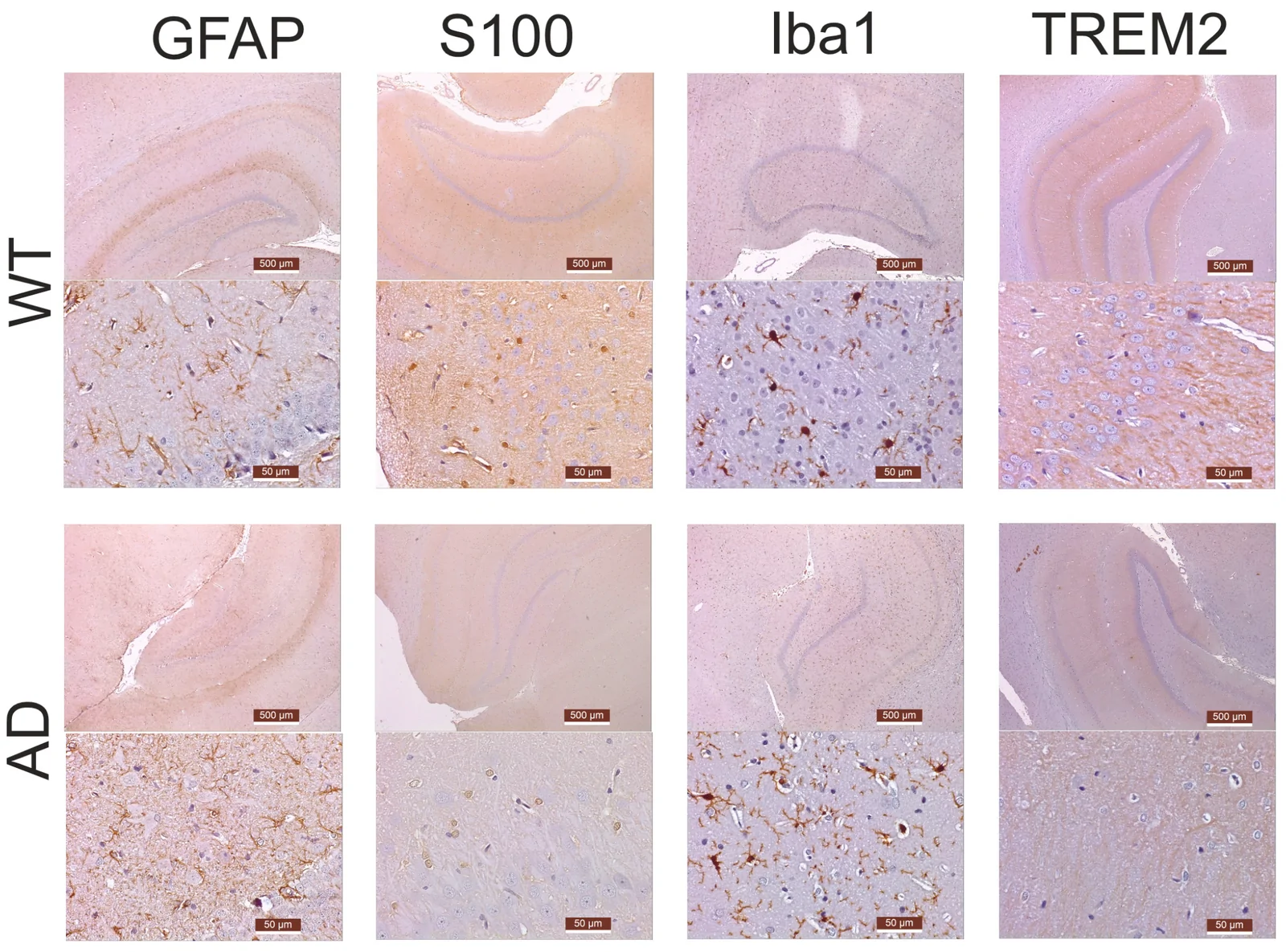

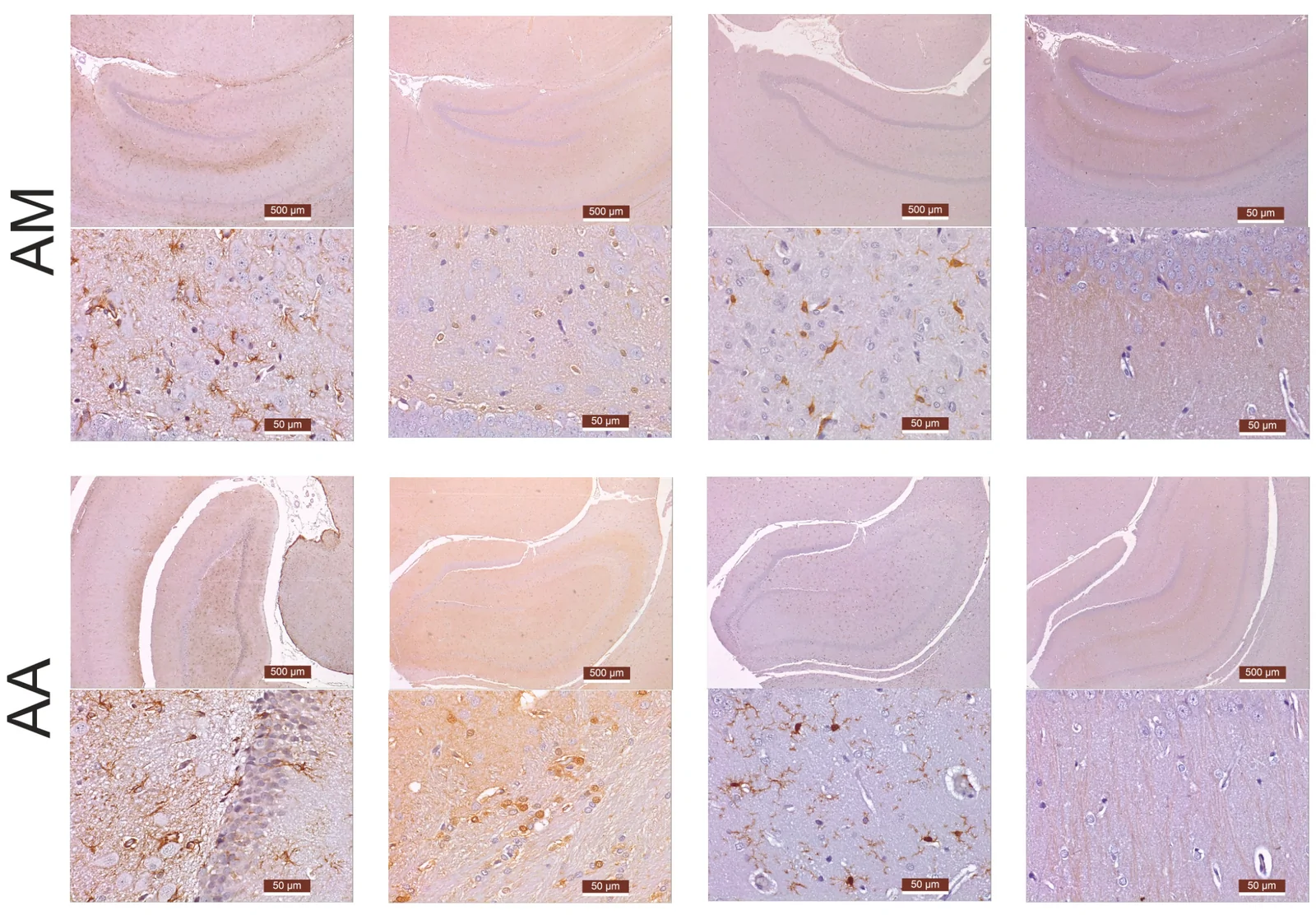
Representative immunohistochemical staining of hippocampal sections for astrocytic and microglial markers across experimental groups. Rows: WT (wild-type controls), AD (Aβ_1–42_-induced Alzheimer’s disease model), AM (AD + intrahippocampal microglial transplantation), and AA (AD + intrahippocampal astrocyte transplantation). Columns: GFAP and S100 (astrocytic markers, reflecting reactive astrogliosis) and Iba1 and TREM2 (microglial markers, reflecting microglial activation/reactivity). For each marker, low-magnification hippocampal overviews (scale bar, 500 µm) are shown above higher-magnification fields (scale bar, 50 µm) illustrating cellular morphology.

Figure 3 illustrates representative immunohistochemical staining of hippocampal sections for astrocytic and microglial markers across experimental groups. In WT animals, GFAP-positive astrocytes appeared sparse, with thin and long processes, and Iba1-positive microglia showed a ramified morphology with fine branches. In AD animals, GFAP-positive astrocytes appeared more numerous and hypertrophic, with thicker processes forming a denser network, and Iba1-positive microglia showed shorter, thicker processes. In the AM group, astrocytic hypertrophy was less evident, and the GFAP-positive network was less dense than in AD animals, while TREM2 staining was more intense in the neuropil. In the AA group, GFAP-positive staining was more frequent, whereas TREM2 staining was reduced. These observations are qualitative and were not quantified at the level of individual cells.

## 3. Discussion

Our findings indicate that transplantation of glial cells, both astrocytes and microglia, improves longitudinal cognitive performance in an amyloid-induced rat model of AD. The effect was more pronounced in the microglial-transplanted group (AM) in the Y-maze, whereas in the NOR test the two treated groups did not differ significantly. Sex subgroup analysis points out a better response for males than females in the Y-maze analysis. Transplantation was performed after confirmation of cognitive impairment, and no adverse effects were detected in the parameters we monitored (body weight and serum IL-6 and TNF-α). These measures are, however, not sufficient to establish the overall safety of the procedure.

A key methodological aspect of our experiment is the longitudinal design in which every animal has its own behavioral control, in addition to comparison with the wild-type and AD groups. Compared to conventional cross-sectional studies, it reduces inter-animal variability and increases statistical power [23,24], while allowing the assessment of dynamic trajectories rather than isolated endpoint differences. This aspect is relevant as Alzheimer’s disease is a progressive neurological disorder.

Our experimental design compares the cognitive decline in the AD group versus the significant improvement in the treated groups (AA and AM) at different time points. This longitudinal pattern is compatible with an effect that goes beyond a transient symptomatic change, although the follow-up period is too short to establish whether the course of the disease itself was modified. These findings support the hypothesis that transplanted glial cells may have contributed to a sustained reconfiguration of the local neuroinflammatory environment and potentially modulated glia phenotypes. However, further molecular investigations will be necessary to confirm the underlying mechanisms directly.

Microglia and astrocytes are central regulators in the pathogenesis of AD, being involved in neuroinflammatory response, amyloid clearance, synaptic remodeling, and metabolic homeostasis [25,26]. Previous transplantation-based treatments in AD rodent models have either relied on intracerebroventricular delivery or systemic administration of hematopoietic or stem cell-derived progenitors, resulting in diffuse cell distribution and a variable response [15,16,27]. Microglia derived from stem cells have demonstrated successful survival, migration, and amyloid phagocytosis [14,27], while transplanted astrocytes have been shown to internalize amyloid deposits after migration from the injection site [15].

In contrast, the present study explored direct stereotaxic intrahippocampal transplantation of primary microglia and astrocytes in an amyloid-induced rat model. This design enables a more controlled assessment of glia-mediated effects on hippocampal-dependent cognition and allows direct migration to affected areas. The use of both glial cell types allows a direct comparison of their effect.

In the Y-maze, microglial transplantation produced a larger and later improvement than astrocyte transplantation, with the AM group exceeding both control groups at S11. In the NOR test, both treated groups performed better than AD animals at S12 but did not differ from each other, so the advantage of microglia is limited to spatial novelty recognition. This difference between tasks is not unexpected, as spatial novelty recognition depends mainly on hippocampal processing of spatial context, whereas object recognition also relies on the perirhinal cortex [28]. Group differences in total exploration time were stable across sessions and did not decrease over time, and SNR is expressed relative to total exploration, so changes in locomotion or motivation are unlikely to account for the improvement in SNR. At S11, SNR in both treated groups was higher than in AD animals and, in the AM group, also higher than in WT animals. The lower WT value at this session probably reflects reduced novelty-driven exploration after repeated exposure to the same apparatus, so the comparison with WT at S11 should be interpreted with caution. This differential effect may reflect the central role of microglia in amyloid recognition, phagocytosis and synaptic remodeling [29]. While astrocyte transplantation provided cognitive improvement, the stronger late effect observed in the microglial-treated group suggests that direct modulation of the innate immune compartment may exert a more pronounced influence on disease trajectory than metabolic support alone.

The gradual behavioral improvement observed after microglial transplantation may reflect time-dependent changes in hippocampal circuitry, but this was not tested directly.

Nevertheless, while the longitudinal trajectory supports the possibility of circuit-level remodeling, the present study was not designed to directly assess synaptic or molecular mechanisms. Further mechanistic investigations will be necessary to determine whether these effects reflect true disease-modifying processes or sustained functional compensation. Taken together, our findings indicate that targeted intrahippocampal transplantation of primary glial cells, applied after confirmation of cognitive impairment, can influence cognitive trajectories in an established amyloid-induced model.

Subgroup sex-stratified analysis revealed a significant group × session × sex interaction for spatial novelty recognition, indicating that longitudinal treatment effects differed between male and female animals. While the study was not powered to investigate sex-dependent mechanisms specifically, this finding suggests that biological sex may influence the temporal response to glial transplantation. Previous reports have described sex-related differences in neuroimmune signaling and microglial reactivity, which could contribute to differential modulation of synaptic function and cognitive performance. However, given the exploratory nature of this analysis and the absence of direct molecular characterization, these observations should be interpreted cautiously. Future studies specifically designed to address sex-dependent glial responses will be necessary to clarify the underlying mechanisms.

Systemic inflammatory markers showed limited group-dependent modulation. IL-6 levels exhibited a significant effect of session and a significant group × sex × session interaction, whereas TNF-α did not demonstrate significant main or interaction effects. These findings suggest that temporal fluctuations in certain cytokines may occur independently of consistent treatment-driven changes and that sex-dependent inflammatory modulation may be cytokine-specific. Importantly, the absence of robust group-dependent systemic inflammatory changes supports the interpretation that the observed cognitive effects are unlikely to be primarily mediated by generalized peripheral inflammation.

IHC markers are used in preclinical studies to demonstrate the effects of specific glial populations. GFAP and S100 are astrogliosis markers that reveal the harmful effects of amyloid plaques in rat-induced models [30,31]. In our model, GFAP immunoreactivity was increased in AD animals and was lower after microglial transplantation. Immunoreactivity remained elevated after astrocyte transplantation. The higher S100 signal in the AA group may reflect the transplanted astrocytes themselves rather than activation of resident cells. Other studies that used stem cells or cells derived from stem cells show a reduction in astrogliosis after treatment [17].

Iba1 and TREM2 are markers of microglial activity in both resting and active states [32]. Recent papers associate increased TREM2 activity with a reactive, phagocytosis-competent microglial state [33], representing a potential target for evaluation and treatment. In our study, TREM2 immunoreactivity was higher in the AM group and lower in the AA group than in the AD animals. Increased TREM2 is consistent with a reactive microglial phenotype, but it is not direct evidence of phagocytosis. TREM2 is also required for microglial clustering around amyloid deposits, where microglia form a barrier that limits plaque diffusion and neuritic damage [33]. Higher TREM2 expression does not automatically translate into amyloid clearance. Our analysis measured the TREM2-positive area and cannot distinguish transplanted from resident microglia or show uptake of amyloid. This would require co-localization of microglial markers with amyloid or lysosomal markers such as CD68, which was not performed. The reduction in TREM2, together with the lower Iba1 signal, after astrocyte transplantation suggests that the transplanted glial cells may have dampened the local microglial response, but the mechanism remains to be clarified.

Apart from GFAP, AD animals did not differ significantly from WT controls for S100, Iba1 or TREM2, and Iba1 showed only a non-significant decrease. In amyloid-injection models, the microglial response is most prominent around the injected deposits during the first days and weeks after injection [34], whereas brain tissue in our study was collected approximately 11 weeks after Aβ_1–42_ injection. At this stage, the microglial reaction may have subsided or become restricted to the immediate vicinity of the deposits, while astrogliosis persisted. In addition, the percentage of positive area measured over the whole hippocampus may dilute focal changes around the deposits, and Iba1 labels resting as well as reactive microglia, so a change in microglial phenotype does not necessarily increase the Iba1-positive area. A cell-level morphological analysis and a time-course design with earlier sampling would better capture these changes.

Nonetheless, our study presents limitations that need to be addressed in future research. Group size was comparable to prior preclinical transplantation studies but limits statistical power for subgroup analysis. Assuming a large effect (Cohen’s d ≈ 1.2), approximately 12 animals per sex in each group would be required to detect sex-dependent differences with 80% power at α = 0.05. Our study did not include a sham-transplantation group receiving vehicle only, so a contribution of the second surgical procedure or of the injection itself to the observed effects cannot be excluded. Survival, distribution and integration of the transplanted cells were not confirmed, as the cells were not labelled before transplantation. The identity and purity of the cultures were assessed only by morphology and were not quantified, and the cell viability was not formally assessed before transplantation. The study did not include detailed molecular characterization of transplanted glial phenotypes or quantitative assessment of amyloid burden and synaptic markers, limiting mechanistic interpretation. The follow-up period does not allow conclusions regarding the long-term durability of the observed effects.

In conclusion, the present study shows that focal intrahippocampal transplantation of primary glial cells can influence longitudinal cognitive performance in an amyloid-induced model of Alzheimer’s disease. The progressive improvement observed, particularly in the microglia-transplanted group, is compatible with a time-dependent modulation of hippocampal function, which needs to be confirmed in longer studies that include mechanistic endpoints.

## 4. Methods

### 4.1. Animals

The experimental study was approved by the Ethics Committee of “Grigore T. Popa” University of Medicine and Pharmacy of Iasi (nr. 483/28.10.2024) and the Romanian National Sanitary Veterinary and Food Safety Authority (nr. 75/18.12.2024). All animal procedures complied with the ARRIVE guidelines and were conducted in accordance with European Community Guidelines (Directive 2010/63/EU) and Romanian legislation (Law no. 43/2014) on the protection of animals used for scientific purposes.

The experiment was conducted at the Advanced Research and Development Center for Experimental Medicine “Prof. Ostin C. Mungiu” (CEMEX). Animals were housed in individually ventilated cages (IVCs) under standard husbandry conditions, including controlled room temperature (20 ± 4 °C), relative humidity (50 ± 5%), and a controlled light-dark cycle with ad libitum access to food and water.

The animals were kept in laboratory conditions during the 7-day quarantine period and were monitored daily and clinically examined for signs of disease or abnormal behavior. Sprague–Dawley rats (*n* = 32), male and female (6 months, 316 g mean weight) were obtained from the Cantacuzino National Institute of Research and Development for Microbiology and Immunology (Bucharest, Romania). Rat pups (p0) (*n* = 3) were used as donors for astrocyte and microglial isolation.

Alzheimer’s disease was induced by stereotaxic intracerebral injection of pre-aggregated amyloid-β_1–42_ peptide, using the same methodological approach previously described by our group. Briefly, Aβ_1–42_ (MedChemExpress) was dissolved in phosphate-buffered saline (PBS, pH 7.4), incubated at 37 °C for 7 days to obtain insoluble fibrillar aggregates, and injected bilaterally into the hippocampus at a dose previously validated to induce stable cognitive impairment without significant mortality. Behavioral assessments were initiated only after model validation, allowing sufficient time for amyloid deposition and the establishment of cognitive deficits before therapeutic intervention [35].

### 4.2. Experimental Design and Timeline

**Figure 4:**
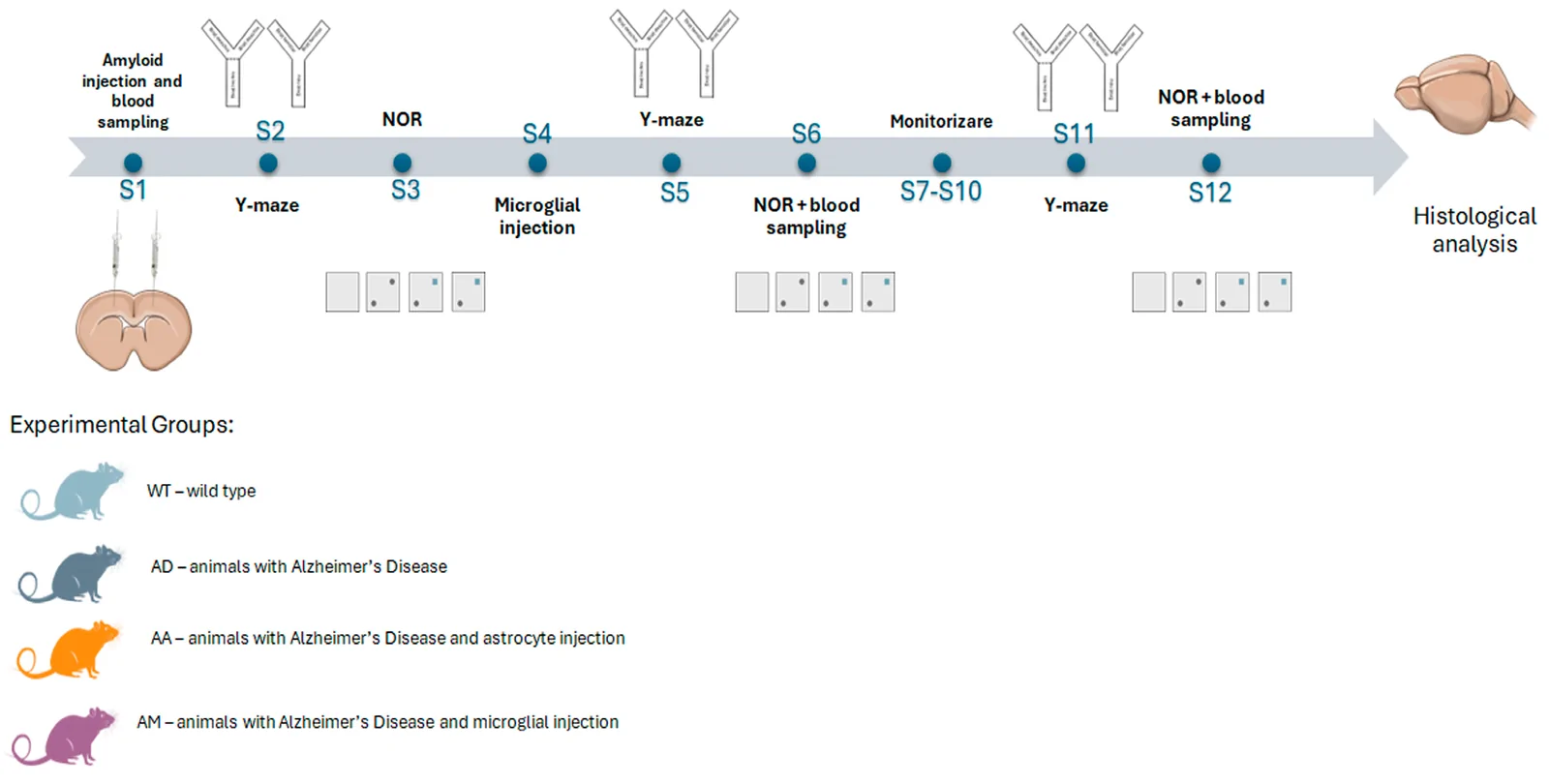
Schematic representation of the experimental design, indicating the time points for Alzheimer’s disease induction, microglial and astrocyte transplantation, behavioral testing, blood sampling, and tissue collection. Each time point (S1–S12) corresponds to one week of the protocol, with one week between consecutive time points; Amyloid injection was performed at S1 and the final evaluation at S12. Illustrations from NIAID NIH BioArt Source (see Acknowledgements section for details).

The study followed a longitudinal experimental design, and animals were assigned to four groups using simple randomization: wild-type (WT, *n* = 6), AD (Alzheimer’s disease, *n* = 6), treated with astrocyte transplantation (AA, *n* = 10) and treated with microglial transplantation (AM, *n* = 10). Group sizes were unequal by design. The control groups (WT and AD) included the minimum number of animals considered sufficient for comparison, while the treatment groups were enlarged to 10 animals each to preserve statistical power in case of losses after the second stereotaxic procedure. No animals died during the study. An overview of the experimental time points and evaluations is shown in Figure 4.

### 4.3. Isolation and Preparation of Astrocytes and Microglia

Primary astrocyte and microglial cells were isolated from neonatal rats using established and previously published protocols [36,37,38,39]. Briefly, cerebral cortices were dissected under sterile conditions, freed of meninges, enzymatically digested, and mechanically dissociated to generate mixed glial cultures. Cultures were maintained in DMEM supplemented with fetal bovine serum and penicillin/streptomycin. For astrocyte isolation, cells were seeded into poly-D-lysine-coated T75 flasks and maintained at 37 °C under standard culture conditions. The culture medium was replaced every 2–3 days. After reaching confluence and forming a stable astrocytic monolayer, cultures were enriched through sequential shaking to remove non-astrocytic cells, followed by PBS washes and detachment using trypsin-EDTA.

After 7–8 days in vitro, microglial cells were selectively harvested from the floating cell fraction, replated onto poly-D-lysine–coated surfaces, and expanded under standard culture conditions. Only microglia-enriched cultures obtained by this selective harvesting procedure were used for subsequent in vivo transplantation procedures.

Astrocyte and microglial identity were determined on the basis of established primary culture timelines, selective harvesting procedures, and characteristic cellular morphology, as previously described in published protocols. Cell identity was therefore established on morphological grounds only. The purity of the cultures was not quantified by immunocytochemistry or flow cytometry, and cell viability was not formally assessed before transplantation. Cells were visually inspected and counted under phase-contrast microscopy before transplantation, and only cultures displaying typical microglial or astrocytic morphology and growth characteristics were used for in vivo administration [35].

### 4.4. Stereotaxic Astrocyte and Microglial Transplantation

Animals were anesthetized with isoflurane (5% for induction and 2.5% for maintenance) and positioned in a stereotaxic frame. Glial cell transplantation was performed using the same stereotaxic coordinates as those applied for Alzheimer’s disease model induction, according to the Paxinos and Watson rat brain atlas (plate 56; AP −2.78, ML ±2.0, DV −3.6). AA (astrocyte transplantation) group was transplanted with astrocytes and the AM (microglial transplantation) group with microglial cell suspension.

A Hamilton syringe was loaded with the cell suspension (2.5 × 10^5^ cells per hemisphere), suspended in 2 μL, and injected at a constant rate of 0.25 μL/min. Following injection, the needle was left in place for an additional 10 min to allow diffusion of the cell suspension within the brain parenchyma. All surgical procedures were performed by trained personnel using standardized stereotaxic techniques. Animals were monitored postoperatively until full recovery. The needle was then withdrawn slowly, and the surgical site was closed with surgical glue (SURGIBOND^®^, SMI AG, St. Vith, Belgium). Animals were monitored postoperatively and received analgesia using buprenorphine (0.01–0.05 mg/kg, subcutaneously) until full recovery.

### 4.5. Behavioral Assay

Cognitive function was evaluated longitudinally using hippocampus-dependent paradigms, including the Y-maze and Novel Object Recognition (NOR) test. All tests were conducted under standardized environmental conditions by an experimenter blinded to the group allocation.

The Y-maze assessed spatial novelty recognition (SNR) in an apparatus that consisted of three identical arms positioned at 120° relative to each other. During the training session, one random arm was blocked, and animals were allowed to explore the remaining two arms freely for 5 min. Following a 1 h inter-trial interval, the blocked arm was opened, and rats were allowed to explore all three arms for 8 min. The previously blocked arm was designated as the novel arm. Repeated assessments were done at the beginning of the experiment (S2), mid-experiment (S5) and final evaluation (S11). SNR was calculated as the percentage of time spent in the novel arm divided by the total exploration time [40].

Recognition memory was assessed using repeated measures NOR with 1 h and 24 h evaluations. Animals were habituated to the testing arena. The next day, they were presented with two identical objects and left for 5 min to explore. Testing was performed after 1 h and 24 h, with one familiar object replaced by a novel object. Experiments were carried out at the beginning (S3), mid-experiment (S6) and at the end (S12). Exploration time was recorded and used to quantify the discrimination index (DI) as the relative preference for the novel object, calculated as the difference between time spent exploring the novel and familiar object, divided by the total exploration time [28,41].

### 4.6. Blood Sampling

Blood samples were collected from the retro-orbital venous plexus using a sterile hematocrit capillary tube at predefined time points: baseline before amyloid injection (S1), mid-experiment (S6) and at the end of the experiment (S12). Sampling volumes were kept within recommended limits. Blood was centrifuged to obtain serum, which was aliquoted and stored at −80 °C until analysis. All procedures were performed by trained personnel using minimally invasive techniques, in line with ARRIVE 2.0 recommendations, to minimize stress-related effects on systemic inflammatory markers.

Serum concentrations of interleukin-6 (IL-6) and tumor necrosis factor-alpha (TNF-α) were measured using species-specific commercial ELISA kits (Elabscience^®^, Rat IL-6, Cat. No. E-EL-R0015; Rat TNF-α, Cat. No. E-EL-R2856), following the manufacturer’s instructions. All samples were analyzed in duplicate, and cytokine levels were expressed in pg/mL based on standard calibration curves.

### 4.7. Tissue Preparation

A comprehensive post-mortem examination was conducted for all animals, comprising systematic external inspection followed by evaluation of the internal organs for the identification of gross pathological abnormalities. Brain tissue was collected for subsequent histological and immunohistochemical analyses. Immediately after collection, tissue specimens were immersed in 10% neutral buffered formalin and fixed for 24–48 h. Following fixation, specimens were trimmed according to organ-specific anatomical planes and processed according to established histological procedures [42]. Automated tissue processing was performed using an Excelsior™ AS Tissue Processor and consisted of sequential dehydration through graded ethanol solutions, xylene-based clearing and paraffin infiltration. Processed tissues were subsequently embedded in paraffin and sectioned at a thickness of 3–4 μm using a semi-automatic rotary microtome (CUT 5062, SLEE Medical GmbH, Nieder-Olm, Germany).

### 4.8. Immunohistochemistry

Quantitative assessment of immunolabeling for the astrocytic and microglial markers GFAP, S100, Iba1 and TREM2 was performed using the QuPath software (version number 0.5.1). The images were set to Brightfield (H-DAB), and in the region of interest—the hippocampus—pixels were classified as positive for cytoplasm and cytoplasmic extensions for GFAP and Iba1, cytoplasm and nucleus for S100, and cytoplasmic granules for TREM2. The percentage of the area that was positive was defined as the amount of DAB-positive pixels in relation to the total area of the hippocampus that was analysed. The mean of the adjacent histological sections analysed for each animal included in the study was calculated, yielding a single representative final value per individual.

### 4.9. Statistical Analysis

Statistical analyses were performed using BlueSky Statistics (version 10.3.4, BlueSky Statistics, LLC|Worldwide Headquarters|2323 W Dickens Ave, Chicago, IL 60647, USA). Behavioral data are presented as estimated marginal means with 95% confidence intervals derived from the mixed-effects models (Y-maze) or as box plots showing the median and interquartile range (NOR). Normality was assessed using the Kolmogorov–Smirnov test. Behavioral and cytokine data were analyzed using linear mixed-effects models with animal ID included as a random effect and group, session/time, and their interaction as fixed effects. Because each animal was tested repeatedly, this approach accounts for within-subject correlation in the same way as a repeated-measures ANOVA, while tolerating unequal group sizes. For the sex-stratified analyses, sex was added as a fixed factor together with its interactions with group and session. No formal a priori power calculation was performed; group sizes were based on previous preclinical transplantation studies and on the reduction principle of the 3Rs. Type III ANOVA was used to assess fixed effects, followed by Tukey-adjusted post hoc comparisons when appropriate. Significance was set at *p* < 0.05.

Statistical analysis of quantitative immunohistochemical data (% positive area for GFAP, S100, Iba1, and TREM2) was performed using IBM SPSS Statistics software (Version 26.0; IBM Corp., Armonk, NY, USA). Data distribution normality within each experimental group was assessed using the Shapiro–Wilk test, and homoscedasticity across groups was evaluated via Levene’s test of equality of variances. Due to a significant violation of the homogeneity of variance assumption across all markers (*p* < 0.05), largely driven by the unbalanced sample sizes, robust statistical procedures were implemented. Overall differences among the four experimental groups were evaluated using Welch’s ANOVA. Post hoc pairwise comparisons were performed using the Games-Howell test, which accommodates heteroscedasticity and unequal sample sizes. Effect sizes for pairwise comparisons were calculated using Hedges’ g. Data are expressed as Mean ± Standard Deviation. All statistical tests were two-tailed, and a *p*-value < 0.05 was considered statistically significant (* *p* < 0.05, ** *p* < 0.01, *** *p* < 0.001).

## 5. Conclusions

Alzheimer’s disease remains a complex neurodegenerative disease in which amyloid plaques trigger a cascade of processes that involve the glial cell population and produce neuronal dysfunction and marked cognitive decline. Both astrocyte and microglial transplantation improved hippocampus-dependent memory in amyloid-injected rats, but the two cell types differed at the tissue level. Microglial transplantation reduced GFAP immunoreactivity and increased TREM2, whereas astrocyte transplantation did not reduce GFAP and was associated with lower TREM2. The advantage of microglia was seen in the Y-maze but not in the NOR test. These results support further work on glial-based approaches, but the survival of transplanted cells, their mechanism of action and durability of the effect need to be demonstrated before any conclusion on disease modification can be drawn.

## Data Availability

The data presented in this study are available on request from the corresponding author due to an ongoing research project.
